# The effects of spaced versus massed extinction training on extinction retention of conditioned fear learning in male rats

**DOI:** 10.3389/fnbeh.2025.1727468

**Published:** 2025-12-09

**Authors:** Grant W. LeVasseur, Timothy Cilley, Michelle Szewczuk, Shane A. Perrine, Seth D. Norrholm

**Affiliations:** Neuroscience Center for Anxiety, Stress, and Trauma (NeuroCAST), Department of Psychiatry and Behavioral Neurosciences, Wayne State University School of Medicine, Detroit, MI, United States

**Keywords:** anxiety, fear conditioning, extinction learning, fear-potentiated startle, extinction retention

## Abstract

**Introduction:**

Extinction learning of conditioned fear behavior has been used as a translational model to study human fear-, anxiety-, trauma-, and stressor-related disorders and their underlying neurobiology in animal models because the underlying neural processes of extinction learning are fundamental to the most effective clinical interventions for these disorders. Specifically, extinction-based prolonged exposure therapy is the first-line, gold-standard, cognitive behavioral treatment for fear-, trauma-, stressor-, and anxiety-based disorders. However, the ways that parametric differences in methodologies alter extinction learning are still not well understood.

**Methods:**

Therefore, in the current study, we altered the number of days on which an equal number of extinction trials were presented in an extinction of conditioned fear learning-paradigm. As part of this paradigm, we employed fear-potentiated startle as a primary outcome measure of fear responses in adult, male rats. One group received 120 massed extinction trials in 1 day, a second group received 120 extinction trials across 2 days, and a final group received 120 extinction trials spaced across 4 days. We hypothesized that a greater number of days of extinction training would lead to improved extinction retention.

**Results:**

We found minimal differences between groups on the final test of extinction retention, although increased fear behaviors were observed at the start of the second day of extinction training in the 2-day group.

**Discussion:**

These findings have implications with respect to the flexibility of fear extinction methodologies employed as well as to how data generated from chosen paradigms is interpreted.

## Highlights

- Fear-potentiated startle is an effective translational platform to study fear.
- Spaced vs. massed extinction training alters extinction training but not retention.
- Parametric differences can impact data interpretation and translation.

## 1 Introduction

Fear- and anxiety-like behaviors (also termed threat responses; see LeDoux, 2014) are present across species and serve an adaptive purpose to enhance the probability of survival in harmful or dangerous situations. However, debilitating behavioral problems can arise when fear- and anxiety-like behaviors become excessive and maladaptive to the point of clinical pathology. Translational neuroscience-based investigations of threat, fear, and anxiety have employed reliable, laboratory-based methodologies to study underlying biological mechanisms, human disease states, and treatments. For example, classical fear conditioning is a useful tool to study threat, fear, and anxiety-like behaviors, across species, towards a goal of gaining a better understanding of psychiatric conditions such as post-traumatic stress disorder (PTSD) and specific phobias (Milad and Quirk, 2012; Maren and Holmes, 2016). Fear conditioning (i.e., forming an association between a neutral stimulus and an aversive unconditioned stimulus following repeated pairing) and fear extinction (i.e., inhibition of the association between the previously neutral, and now conditioned stimulus, and the aversive stimulus) protocols allow for the examination of different phases of learning that regulate the expression of threat, fear, and anxiety-like behaviors. Research has shown that in *de novo* fear conditioning protocols, PTSD patients show impairments in extinction learning, including extinction retention (a.k.a. extinction recall) that can be rescued with prolonged exposure therapy treatment (Norrholm et al., 2011; Norrholm and Jovanovic, 2018; Maples-Keller et al., 2022). Extinction learning processes are fundamental to prolonged exposure therapy, which is a first-line, evidence-based, and gold standard cognitive behavioral treatment for fear-, trauma-, stressor-, and anxiety-related disorders (Rothbaum and Davis, 2003; Craske et al., 2008).

Extinction learning provides a translational approach to study fear learning, however, differences in methodological approaches may influence how pre-clinical data is interpreted, which may, in turn, have implications for its clinical applications. For example, the effects of altering the interval (on the order of minutes to days) between acquisition training and extinction training on long term extinction have been explored (Rescorla, 2004; Maren and Chang, 2006; Myers et al., 2006; Woods and Bouton, 2008). These studies found that the long-term extinction effects varied depending on the outcome measure of fearful behavior, with a fear-potentiated startle (FPS)-based protocol showing shorter acquisition-extinction intervals (i.e., a shorter time period between the phases of fear learning) were associated with significantly lower conditioned fear after extinction training (i.e., extinction retention), whereas studies using freezing as the outcome measure found the opposite result (Rescorla, 2004; Maren and Chang, 2006; Myers et al., 2006; Woods and Bouton, 2008). A parametric discussion of FPS versus freezing as an index of threat, fear, or anxiety-like behaviors is beyond the scope of the current study and has been discussed elsewhere (Rodgers, 1997; Rosen and Schulkin, 1998; Fendt and Fanselow, 1999; Pare et al., 2004). For the current study, we employed FPS for two primary reasons: (1) FPS possesses high translatability across species due to similar methods being used (e.g., cue presentation, response observation and quantification) and neurobiological (e.g., homologous tri-synaptic reflex circuit) overlap and (2) very few rodent studies have used FPS to explore how variations of extinction learning parameters affect extinction retention. At present, FPS is one of the primary means with which extinction learning is studied in pre-clinical and clinical human studies (Norrholm and Jovanovic, 2018). However, much of the animal data informing these human studies has been based on a heterogeneous body of literature using methods not directly applicable to humans (e.g., freezing). Therefore, the current study aims to address that translational gap by exploring spaced versus massed extinction in a manner directly translatable to human investigations using a fear index that is highly conserved across species.

Decades of research on learning theory, and extinction studies specifically, have highlighted the benefits of spaced versus massed learning (i.e., fewer extinction learning trials over a few days versus a lot of trials on a single day) (Bloom and Shuell, 1981; Cepeda et al., 2006; Rohrer and Pashler, 2007; Litman and Davachi, 2008). Some reports have shown that spaced extinction training with longer intertrial intervals (ITIs; i.e., the period of time between the presentation of two conditioned cues) leads to improved long-term extinction retention learning compared to massed extinction training with shorter ITIs (Li and Westbrook, 2008; Urcelay et al., 2009). An earlier report (Cain et al., 2003) found the opposite result: shorter massed ITIs in a single session led to improved extinction retention, but interestingly presenting the CS over several days led to improved retention compared to a single massed session.

Two studies using an appetitive conditioning task found that an increased number of extinction sessions decreased response renewal (Nieto et al., 2023) and increasing the time between extinction sessions decreased spontaneous recovery (Tapias-Espinosa et al., 2018). Furthermore, multiple days of extinction training blocked spontaneous recovery of fear (Rossato et al., 2010) and increasing the number of days of training improved the extinction learning response (Walker et al., 2002). A recent study by Gerhard and Meyer (2021) explored this concept in adult and adolescent mice by performing extinction training over 1, 2, or 4 days. They found that spaced and massed extinction training resulted in similar levels of retention in adolescents, whereas only spaced extinction trials improved extinction retention in adults (Gerhard and Meyer, 2021). The goal of the present pre-clinical study was to explore how spaced (multi-day) versus massed (single session) extinction training affects within-and between-session extinction learning in a FPS-based fear conditioning task. We hypothesized that presenting extinction training over spaced sessions, compared to a single massed session, would facilitate retention of extinction learning when tested on a separate day.

## 2 Methods

### 2.1 Animal subjects

**Figure 1:**
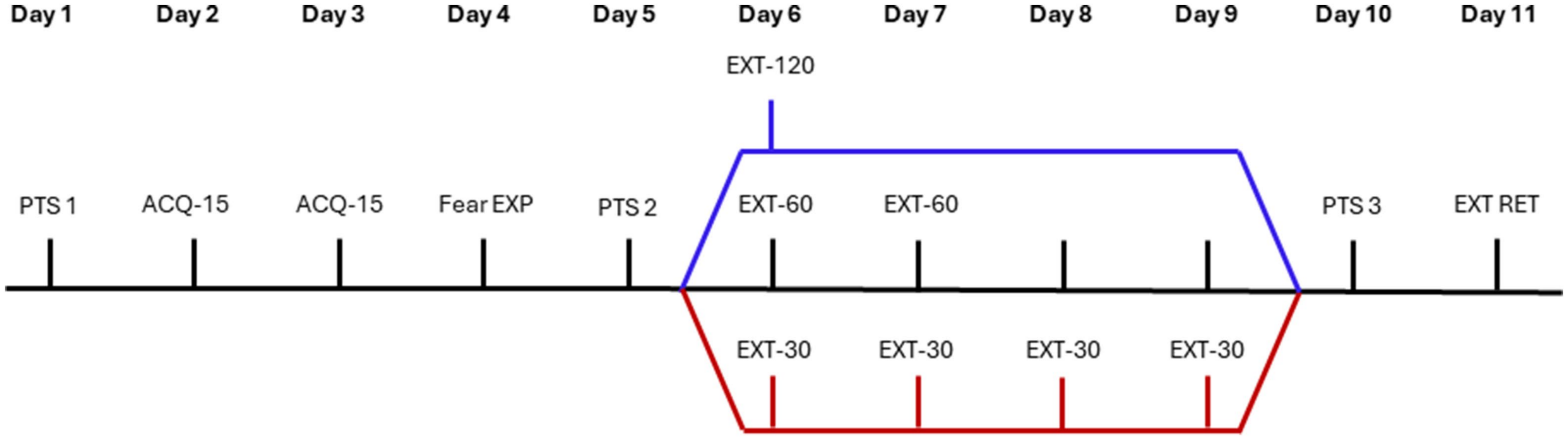
Experimental design. Rats were divided into three experimental groups, based on extinction training type: massed extinction (blue trace), in which rats underwent a single extinction session with 120 CS presentations, 2-day spaced extinction (black trace), in which rats underwent two extinction sessions with 60 CS presentations on each day, or 4-day spaced extinction (red trace), in which rats underwent four extinction sessions with 30 CS presentations on each day. PTS, pre-test startle; ACQ, acquisition; EXP, expression; EXT, extinction; RET, retention.

Twenty-four (24) male Sprague Dawley rats (>postnatal day 60) were purchased from Charles River Laboratories (Raleigh, NC) for use in the current experiments. This number of rats was chosen based on our groups’ previous work (Young et al., 2017), using similar methods. The rats were transitioned to a reversed light–dark cycle (lights off at 06:00, lights on at 18:00) upon arrival and allowed to acclimate for 5 days before experimentation, as previously reported by our group (Gheidi et al., 2023). The rats were pair housed for the duration of the experiment in standard rat cages and housing conditions with a reversed light–dark cycle to minimize the impact of sleep disturbances on behavioral outcomes and align experimenter and rodent active cycles. Animals were provided standard rat chow and water *ad libitum* except during fear conditioning sessions. All rats were handled by the experimenter for 1–2 min per day for 2 days before the start of behavioral tests to habituate the rats to the experimenter. Behavioral experiments began 5 days after arrival and rats weighed 326 g ± 3.9 g (mean ± SEM). All procedures were performed during the animals’ active phase (between 08:00 and 17:00) under dim red light. Before each learning test, rats were given 30 min to habituate to the behavioral room. Importantly, the rats were split into three groups based on the type of extinction training they would receive: 1-day (*n* = 5), 2-day (*n* = 8), or 4-day (*n* = 7) (see Figure 1). All groups began with eight subjects, but four rats were excluded from analysis due to a failure to reach a fear acquisition learning criterion defined as a fear-potentiated startle response during the fear expression test greater than 30% compared to baseline; a criterion based on applications by Myers and Davis (2004). All experimental procedures were approved by the Wayne State University Institutional Animal Care and Use Committee under approval number IACUC-18-10-0810.

### 2.2 Fear learning tests

Eight SR-LAB startle chambers (San Diego Instruments, San Diego, CA) were used simultaneously to conduct this experiment. Each chamber is a sound attenuating box with the capacity to provide audio and visual stimuli via speakers and cue lights, respectively. Within each chamber, a clear, polyacrylic, cylindrical startle enclosure is placed with each chamber, containing a piezoelectric sensor attached to the bottom. The chambers and enclosures are all linked to a closed-loop computer system which can deliver stimuli and record animal startle behavior simultaneously. All piezoelectric sensor outputs were standardized to one another before behavior tests each day using a standardization unit (San Diego Instruments). All startle enclosures were cleaned with Peroxigard™ before and after each use between rats.

#### 2.2.1 Pre-test startle assessments

Rats were assessed for differences in their baseline startle response via pre-test startle (PTS) measurements. Pre-test (baseline) startle levels were assessed at 3 separate time points throughout this study: before the start of fear conditioning, before the start of extinction training, and before the start of extinction retention testing. For PTS, rats were placed in separate startle enclosures and exposed to 50 dB background white-noise. After a 300 s habituation period, the animals were exposed to 30 white-noise startle probes (40 ms, 100 dB). Each startle probe (SP) was separated by a 30–90 s intertrial interval (ITI). Startle amplitude was defined as the maximum voltage output (in mV) from the accelerometer recorded during a 100 ms window after the onset of the SP. Rats were returned to their home cages shortly after the termination of the final SP. Throughout the rest of this text, the trials in which the startle probe is presented in the absence of any other stimuli are referred to as “Noise Alone” (NA) trials.

#### 2.2.2 Fear acquisition session

Olfactory and visual elements of context were included in the current set of experiments. For Context A, a piece of filter paper soaked with 2.5% acetic acid was placed in each startle chamber, the walls of the startle chamber were kept a blank white, and the startle enclosures were fitted with metal shock bar inserts. The conditioned stimulus (CS) was a white light, and the unconditioned stimulus (US) was a cutaneous footshock. For fear acquisition, the rats were placed in separate startle enclosures in Context A and exposed to 50 dB background white-noise. After a 300 s habituation period, the animals were exposed to 15 light-shock (CS-US) pairings with an ITI of 30–90 s (60 s average). A 0.6 mA shock US was delivered during the last 500 ms of the 3,700 ms presentation of the light cue. Each rat underwent two fear acquisition sessions separated by 24 h, for a total of 30 light-shock pairings.

#### 2.2.3 Fear expression test

Rats were returned to Context A to test for the expression of the conditioned FPS response (CR). After 300 s of habituation with 50 dB background white-noise, the rats were presented with 10 NA trials to establish a startle baseline, followed by the presentation of a mixture of “Light-Noise” (10) or “Noise Alone” (10) trials in a pseudorandom order. Light noise trials consisted of co-presentation of the 3,700 ms light cue and startle probe, such that the startle probe was presented at 3200 ms, matching the time that the shock was presented in the acquisition trials. The “Light Noise” and “Noise Alone” trials were ordered in such a way that no more than 3 of the same type were presented in a row. There was a 30–90 s (60 s average) ITI between trials. No shocks were administered during this phase.

#### 2.2.4 Fear extinction training

Fear extinction training occurred in Context B which contained filter papers soaked with 2.5% lemon essential oil solution and a black/white checkerboard pattern on the chamber walls. After 300 s of habituation with 50 dB background white-noise, the rats were presented with (unreinforced) light cues without being shocked. All rats received 120 extinction trials with a 30–90 s ITIs (60 s average). The number of trials presented within a single session depended on the experimental group of the rat, but the total time of extinction learning was equal (2 h) in all groups. There was a 300 s habituation period before each daily session. Rats in the 1-day group received 120 extinction trials in a single massed session. Rats in the 2-day group received 60 extinction trials in each daily session. Rats in the spaced 4-day group received 30 extinction trials in each daily session. No “Noise Alone” trials were presented during extinction training. Extinction response was calculated as % FPS using the average startle response from PTS 2 and PTS 3 as a baseline.

#### 2.2.5 Fear extinction retention test

The rats were returned to context B 24 h after the final PTS session and were presented with cued and uncued (noise alone) startle probes in a manner similar to the fear expression test. After 300 s of habituation with 50 dB background white-noise, the rats were presented with 10 NA trials to measure PTS baseline. The rats were then presented with a mixture of “Light-Noise” (10) or “Noise Alone” (10) trials in a pseudorandom order in the same manner as during the fear expression test.

### 2.3 Statistical methods

PTS and fear expression assessments were measured using startle magnitude, which was defined as the maximum mV response recorded during the 100 ms window after presentation of the startle probe divided by the weight of the animal during that test day [similar to methods previously employed (Olivera-Pasilio and Dabrowska, 2023)]. Fear extinction training, and extinction retention were analyzed using percent startle potentiation, calculated using the following equation: [((cued − uncued)/uncued)*100]. Cued refers to Light-Noise trials while uncued refers to Noise Alone trials.

Four rats were excluded from analysis because they failed to show at least 30% startle potentiation during the fear expression test. This was done because we were primarily interested in the process of extinction, which is difficult to study if the subjects do not show sufficient acquisition of fear in the first place. An additional subject in the 2-day group was excluded from the analysis of extinction training because their startle response was more than 3 standard deviations above the mean for the group, but the response in other phase of learning was within the expected range. For analysis of extinction data, startle responses were averaged across 10 extinction trials and reported as extinction epochs. One- or two-way ANOVAs were performed on the data generated in this study, depending on comparison of interest. When necessary, multiple individual comparison tests were performed among groups analyzed via ANOVA using Tukey’s multiple comparison test. Geisser–Greenhouse corrections were used in analyses in which there were violations of the assumption of sphericity.

All statistical analyses were performed using GraphPad Prism version 10.3.1 (509) (GraphPad Software, Inc., San Deigo, CA). *p < 0.05* was considered significant.

## 3 Results

### 3.1 Pre-test startle assessments

**Figure 2:**
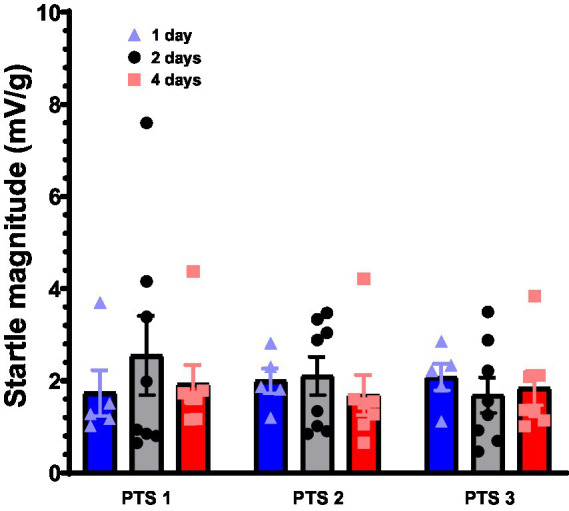
Pre-test startle assessments. Rats received 30 NA trials before the start of fear conditioning (PTS 1), before the start of extinction training (PTS 2) and before the start of the final extinction retention test (PTS 3). Maximum startle amplitude for each animal was collected and was standardized to each animal’s body weight and this value was recorded as startle magnitude. Startle magnitude was averaged for the entire experimental group across 30 trials and is displayed above for each separate test day. Error bars represent SEM.

The mean startle magnitude during PTS 1 was 1.74 mV/g for the 1-day group, 2.55 mV/g for the 2-day group, and 1.93 mV/g for the 4-day group. The mean startle magnitude during PTS 2 was 2.00 mV/g for the 1-day group, 2.10 mV/g for the 2-day group, and 1.69 mV/g for the 4-day group. The mean startle magnitude during PTS 3 was 2.08 mV/g for the 1-day group, 1.69 mV/g for the 2-day group, and 1.84 mV/g for the 4-day group. A two-way ANOVA was performed on this data with a Geisser–Greenhouse correction due to violations of the assumption of sphericity. Two-way ANOVA revealed no significant difference between groups, nor a significant difference among test days (see Figure 2). There was no significant interaction effect between extinction training group and PTS test day (*F* (4, 34) = 0.98, *p* = 0.43). There was no main effect of extinction group (*F* (2, 17) = 0.13, *p* = 0.88) or test day (*F* (1.3, 22) = 0.31, *p* = 0.64).

### 3.2 Fear expression test

**Figure 3:**
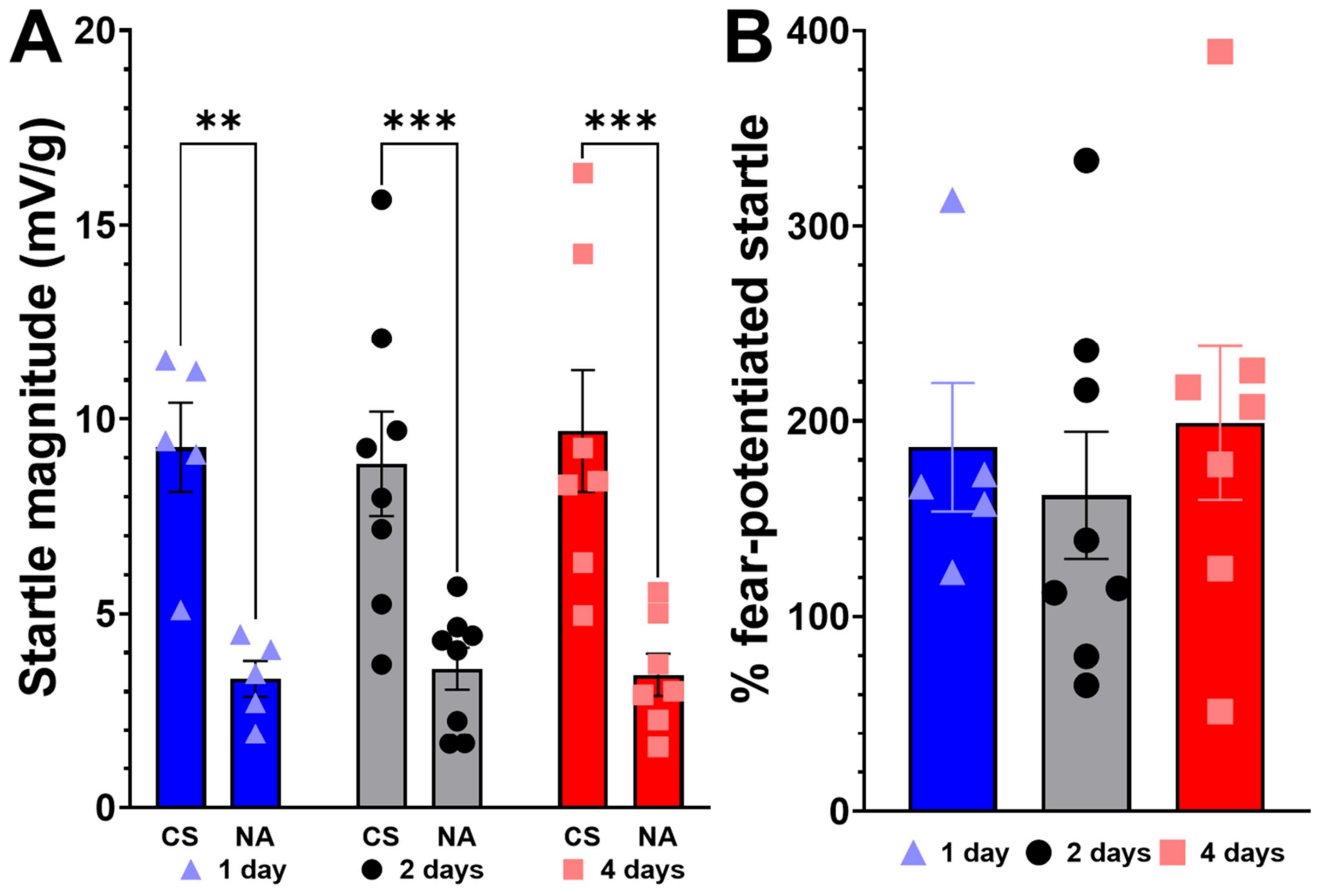
Expression of fear. Fear-potentiated startle (FPS) to the CS (chamber light) was measured in Context A (acquisition context) 24 h after completion of the second fear acquisition training session. **(A)** Displays the startle magnitude, **(B)** Displays the % FPS. Bars labeled as CS represent the average startle magnitude during the 10 cued trials. Bars labeled as NA represent the average startle magnitude during the 10 uncued trials. The 1 day extinction group (*n* = 5) is shown in blue, triangles indicate individual subject data. The 2 day extinction group (*n* = 8) is shown in grey, with circle subject markers. The 4 day (*n* = 7) extinction group is shown in red, with square subject markers. Two-way ANOVA revealed no significant effect of extinction group, but a significant effect of cue presentation. Multiple comparisons revealed a significant difference between cued and uncued presentations in all groups, marked by **p < 0.01, ***p < 0.001 above each comparison. Values are expressed as group mean ± SEM.

The rats in all 3 groups displayed successful and similar levels of acquisition of fear on the fear expression test (see Figure 3). Successful fear acquisition was determined to be a greater than 30% potentiation of startle in response to the conditioned cue compared to uncued (noise alone) trials, and only 4 rats were removed from further analyses for not meeting this threshold. A two-way ANOVA of startle magnitude (Figure 3A) revealed a main effect of cue presentation (*F* (1, 34) = 42.2, *p* < 0.0001) but no main effect of extinction group (*F* (2, 34) = 0.06, *p* = 0.94) and no interaction effect (*F* (2, 34) = 0.13, *p* = 0.88). Multiple comparisons were made within each extinction group between cued and uncued trials using Tukey’s multiple comparison test. A one-way ANOVA of % FPS (Figure 3B) revealed no significant differences among groups (*F* (2, 17) = 0.31, *p* = 0.74); the expression data are presented as % FPS to maintain consistency of measurement throughout this section. The 1-day group had an average startle magnitude of 9.27 mV/g and 3.33 mV/g in response to the CS and NA trials, respectively, (*p* = 0.002), and showed an average potentiation of 187% (percent potentiation was calculated separately for each animal and then averaged). The 2-day group had an average startle magnitude of 8.85 mV/g and 3.59 mV/g in response to the CS and NA trials, respectively, (*p* = 0.0006), and showed an average potentiation of 137%. The 4-day group had an average startle magnitude of 9.69 mV/g and 3.43 mV/g in response to the CS and NA trials, respectively, (*p* = 0.0002), and showed an average potentiation of 199%.

### 3.3 Fear extinction training

**Figure 4:**
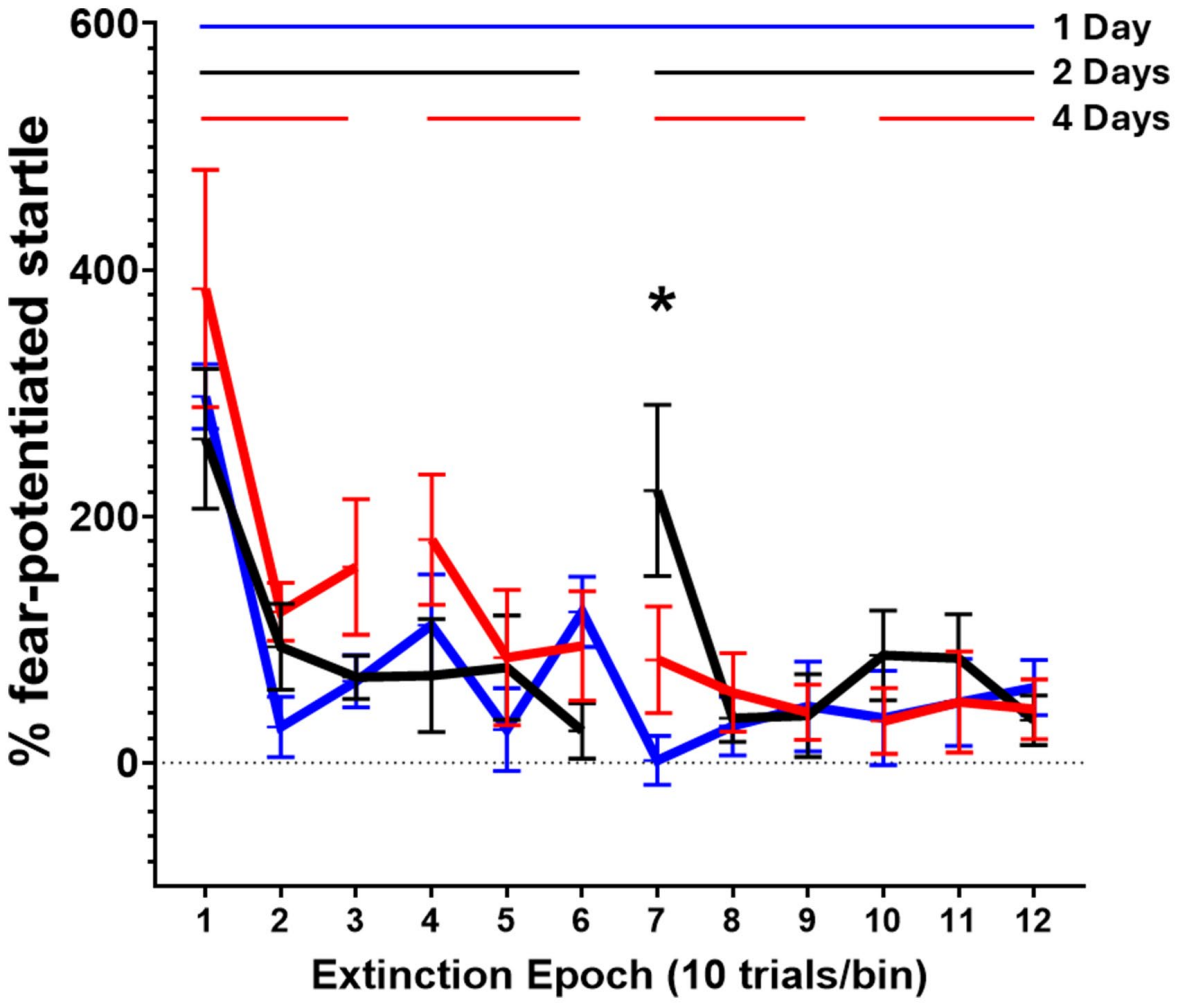
Extinction training. Rats were exposed to 120 presentations of the CS in Context B. The number of sessions across which these presentations were spread differed depending on the group. Traces show average % FPS of 10 CS presentations. Rats that received extinction training in a single session over 1 day (*n* = 5) are shown with the continuous blue trace. Rats that received extinction training over 2 days (*n* = 7, 60 CSs/session) are shown with the fragmented black trace. Rats that received extinction training over 4 days (*n* = 7, 30 CSs/session) are shown with the fragmented red trace. * Indicates a significant difference (*p* < 0.05) between the 2 day group and the 1 day and 4 day groups. Values are expressed as group mean ± SEM. Extinction data from 1 rat in the 2 day group was excluded as an outlier.

All three groups exhibited successful within-session extinction learning, as determined by a significant decrease in startle potentiation from the start to the end of extinction training (see Figure 4). Two-way ANOVA was performed on the data from the 12 epochs of extinction training. There was a significant interaction between extinction group and extinction epoch (*F* (22, 176) = 1.73, *p* = 0.02), meaning that the manner in which rats exhibited startle during the 120 trials of extinction training varied among the 3 different groups. There was a main effect of extinction epoch (*F* (11, 176) = 12.2, *p* < 0.0001) but no main effect of extinction group (*F* (2, 16) = 0.67, *p* = 0.53). Multiple comparisons were performed within each extinction epoch among extinction groups using Tukey’s multiple comparisons test. The 2-day group exhibited a significant increase in startle during the 7^th^ epoch (start of day 2) compared to both the 1-day group (*p* = 0.001) and the 4-day group (*p* = 0.04).

### 3.4 Fear extinction retention test

**Figure 5:**
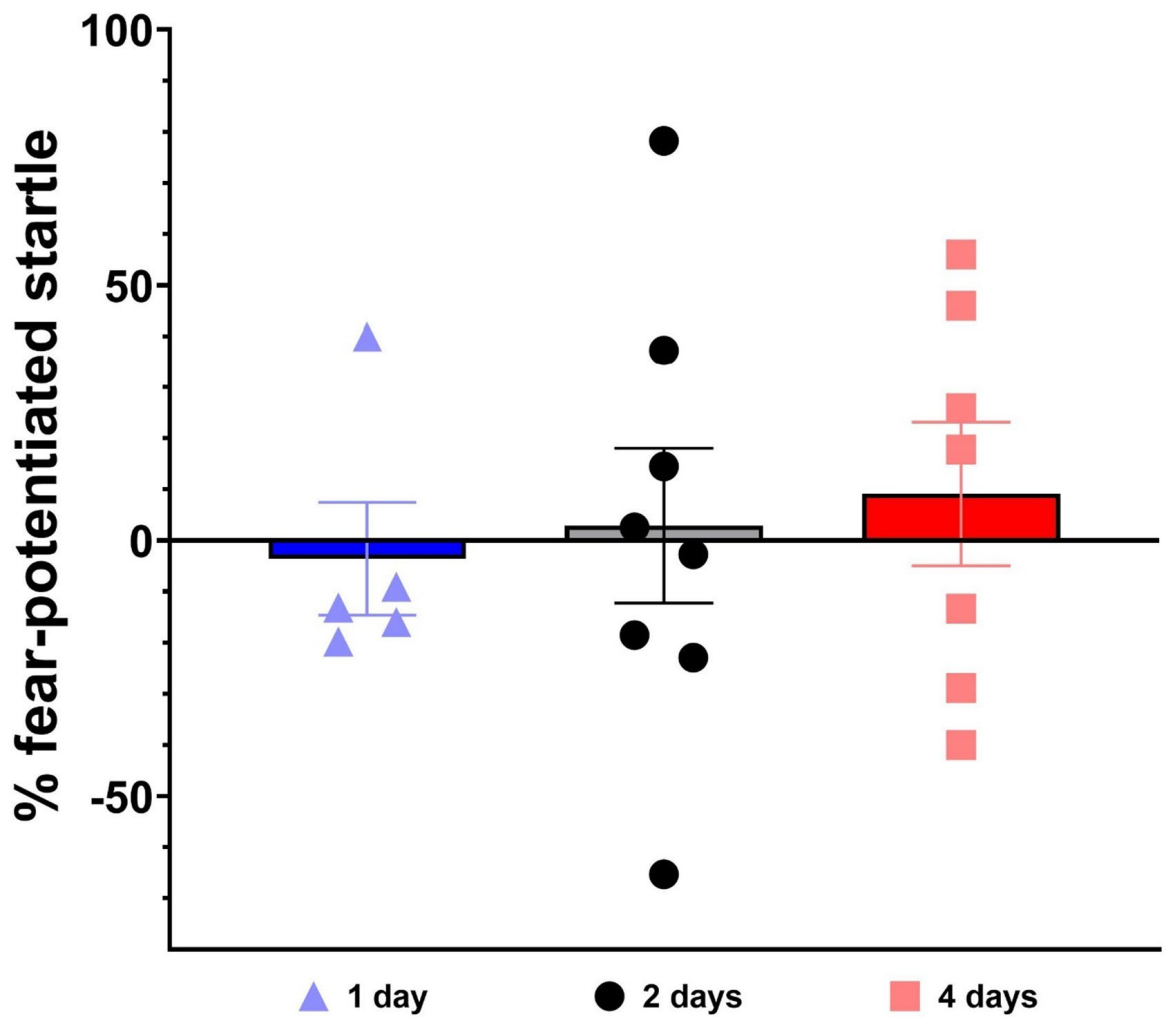
Test of extinction retention. FPS to the CS was measured in Context B after the completion of extinction training. % FPS was calculated as the percent difference in mean startle during CS trials compared to mean startle during 10 NA trials. The 1 day extinction group (*n* = 5) is shown in blue, triangles indicate individual subject data. The 2 day extinction group (*n* = 8) is shown in grey with circle subject markers. The 4 day (*n* = 7) extinction group is shown in red with square subject markers. One-way ANOVA reveals no significant difference between groups. Values are expressed as group mean ± SEM.

All three groups of rats exhibited successful between-session extinction retention, indicated by a lack of startle potentiation in response to the extinguished CS (see Figure 5). The rats in the 1-day group had a mean startle potentiation of −4%, the rats in the 2-day group had a mean startle potentiation of 3%, and the rats in the 4-day group had a mean startle potentiation of 9%. One-way ANOVA revealed no significant difference between groups, indicating there was no significant effect of extinction group type on the results of the extinction retention test (*F* (2, 17) = 0.17, *p* = 0.84). Repeated measures ANOVA revealed no significant interaction between extinction group and fear learning phase upon comparison of mean startle potentiation from the 12^th^ epoch of extinction training (end) and the extinction retention test (*F* (2, 33) = 0.39, *p* = 0.68).

## 4 Discussion

The present study assessed the impact of spaced and massed extinction training on a final test of extinction retention measured using FPS. We show that altering the number of days over which extinction training is presented did not affect extinction retention. All groups displayed successful extinction retention, regardless of the number of days of extinction training (see Figure 5). These results did not support our hypothesis that spaced extinction learning would facilitate extinction retention. However, differences were observed among groups during extinction training. Namely, there was an increase in FPS in the 2-day extinction group at the start of day 2 of extinction training (epoch 7), compared to the 1-day and 4-day groups (see Figure 4), after displaying a nearly fully extinguished FPS response at the end of day 1 (epoch 6). Such differences may be expected, according to the Rescorla-Wagner model of reinforcement learning, and arise due to variations in expectancy between groups stemming from the differing temporal contexts of learning in each group at this time point (Rescorla and Wagner, 1972). Maintaining the session and context for the 1-day massed extinction group leads to lower expectancy of an aversive stimulus during epoch 7, and the consolidation of learning from two previous days without an aversive stimulus keeps expectancy low in the 4-day spaced extinction group. In other words, the extinction memory is likely dominating the acquisition memory in the 4-day spaced extinction group at this time point due to repeated consolidation over days. Expectancy of an aversive stimulus may be anticipated to rise at epoch 7 of extinction in the 2-day spaced extinction group due to weaker consolidation of the extinction memory, and substantial (lingering) recall of the original acquisition memory. However, it was surprising to us that we did not observe a similar increase in FPS on day 2 of EXT training in the 4-day group (epoch 4). Such an increase (or possibly a greater increase) would be expected, based on our posited explanation. The lack of this observation may be due to the fact that the FPS response was not fully extinguished in the 4-day group at the end of day 1 (epoch 3) and that this non-extinguished FPS response was simply maintained at the start of day 2 (epoch 4). Individual differences in extinction learning and high variability in FPS within this cohort may also be a factor. There is a slight upward trend in the extinction curve for the 4-day group at epoch 4, and the 4-day group has the highest rank order when comparing mean FPS at this time-point, but neither of these comparisons reach the level of statistical significance. Observing such differences underscored the usefulness of spreading extinction training out over multiple days to observe differences or deficits in extinction learning that may be present in the subjects but are obscured by administering extinction in a single massed session.

Previous literature has demonstrated that delivering extinction trials over several days leads to improved extinction retention (Cain et al., 2003; Gerhard and Meyer, 2021). The results of the present study conflict with these previous results, although we observed notable extinction retention in all groups. We did not significantly vary the ITI between extinction trials as a form of massing or spacing as others have done (Cain et al., 2003; Li and Westbrook, 2008; Urcelay et al., 2009) with mixed results. The intention of the present study was to assess the effects of trial spacing across days, similar to the spacing used by Gerhard and Meyer (2021), who also used a different measure of fear and advocate for the use of multiple fear indices (Gerhard and Meyer, 2021). Although the results of the present study conducted using FPS show no differences between massing or spacing trials over days, we do not believe that FPS is a wholly unique measure of fear, but rather that the regulation of fear is a dynamic process and that different fear indices can help capture differential aspects of this process.

There were some limitations of note in this present study. First, this study was only done in male rodents. It is well known that the process of extinction learning is affected by sex differences including fluctuating estrogen levels, so it will be important to study these same effects in female rodents in the future (Graham and Milad, 2013; Glover et al., 2015). The present study is intended to be a parametric analysis of factors that affect extinction learning and was limited to males. Second, the timing of extinction training in relation to the final test of extinction retention could have been altered. In the present study, rats in the 1-day and 2-day groups had an extra 3 or 2 days, respectively, of incubation time in which no tests were performed before the final test of extinction retention. This may have impacted the results of the final test, but we chose to prioritize starting extinction training at an equal time after acquisition learning in order to maintain a high level of fearful behavior at the start of extinction in all groups. A report by Quirk (2002) shows retention of the extinction memory on a test of spontaneous recovery up to 6 days following extinction training, a period of time greater than the interval between extinction training and retention testing employed in our study (Quirk, 2002). Presently, it is unclear to us whether this low level of fear during extinction retention testing would be maintained over a longer period of time (spontaneous recovery) or maintained in a new context (fear renewal). Such tests may have unmasked differences among these groups, however, the focus of this study was to examine short-term within- and between-session extinction. Third, it is possible that including 120 extinction trials may have resulted in “over extinction” and a floor effect that may have masked differences in the extinction retention test. Fourth, the number of CS-US pairings (30) is high, but not out of the realm of published literature (Groenink et al., 2023) and the nature of the fear expression test makes it a *de facto* extinction training session (although one would expect this to occur later in the expression test session). Because these parameters were kept constant for all groups in this study, we do not believe these parameters had a major effect on the outcome of this study. Lastly, we chose not to include control groups for fear conditioning (no shock) and extinction because Pavlovian fear learning is a well-established method, although these controls may have strengthened our conclusions (Rescorla, 1968; Phillips and LeDoux, 1992; Chhatwal et al., 2005). Despite these limitations, this study revealed no significant differences between spaced and massed extinction on a test of extinction retention when measured using FPS. This suggests a flexibility in method design, such that the delivery of extinction training and measurement of fearful behaviors may be modified to better answer the question being asked and the outcome measure of interest.

One such difference in methodological approach leading to divergent fear learning outcomes was well summarized by Maren (2014) in regards to the timing of extinction training relative to acquisition training. Rescorla (2004) showed that shorter acquisition-extinction intervals increased the magnitude of spontaneous recovery in an appetitive conditioning task (Rescorla, 2004). In contrast, Myers et al. (2006) used an aversive, FPS based conditioning procedure to show that shorter acquisition-extinction intervals led to a block of spontaneous recovery compared to greater spontaneous recovery seen with longer intervals (Myers et al., 2006). Follow-up studies showed that shorter acquisition-extinction intervals led to weaker long-term extinction with fearful behavior measured via freezing and conditioned response suppression, respectively (Maren and Chang, 2006; Woods and Bouton, 2008).

Overall, we have developed a FPS based conditioning protocol for the purpose of studying extinction learning and have shown that the number of days of extinction training may not impact the way that extinction memories are retained. However, if one is interested in the learning processes underlying the extinction training phase then the methods used to extinguish conditioned fear may have greater relevance and applicability. These data have important clinical implications as they reveal that the translational method of measuring extinction learning may impact any conclusions drawn from such examinations based on the specific protocols employed. This may impact the clinical translation of pre-clinical extinction studies and shows that caution should be taken in assuming the direct translational relevance of any one study, including this study. These data and methodologies will be useful going forward as a translational platform to further study how interventions like pharmacological or stress-evoking manipulations may impact extinction learning.

## Data Availability

The raw data supporting the results and conclusions of this study will be made available by the authors without undue reservation.
